# Optimization of Electroporation Conditions for Introducing Heterologous DNA into *Rhodobacter sphaeroides*

**DOI:** 10.4014/jmb.2408.08044

**Published:** 2024-09-20

**Authors:** Yu Rim Lee, Juah Lee, Suhyeon Hong, Soo Youn Lee, Won-Heong Lee, Minseob Koh, In Seop Chang, Sangmin Lee

**Affiliations:** 1 National Biotechnology Policy Research Center, Korea Research Institute of Bioscience and Biotechnology, Daejeon 34141, Republic of Korea; 2 School of Earth Sciences and Environmental Engineering, Gwangju Institute of Science and Technology (GIST), Gwangju 61005, Republic of Korea; 3 Department of Bio-Environmental Chemistry, College of Agricultural and Life Sciences, Chungnam National University, Daejeon 34134, Republic of Korea; 4 Gwangju Clean Energy Research Center, Korea Institute of Energy Research, Gwangju, 61003, Republic of Korea; 5 Department of Bioenergy Science and Technology, Chonnam National University, Gwangju 61186, Republic of Korea; 6 Department of Chemistry and Chemistry Institute for Functional Materials, Pusan National University, Busan 46241, Republic of Korea

**Keywords:** *Rhodobacter sphaeroides*, transformation, electroporation

## Abstract

*Rhodobacter sphaeroides* is a strain capable of both photoautotrophic and chemoautotrophic growth, with various metabolic pathways that make it highly suitable for converting carbon dioxide into high value-added products. However, its low transformation efficiency has posed challenges for genetic and metabolic engineering of this strain. In this study, we aimed to increase the transformation efficiency of *R. sphaeroides* by deleting the *rshI* gene coding for an endogenous DNA restriction enzyme that inhibits. We evaluated the effects of growth conditions for making electrocompetent cells and optimized electroporation parameters to be a cuvette width of 0.1 cm, an electric field strength of 30 kV/cm, a resistance of 200 Ω, and a plasmid DNA amount of 0.5 μg, followed by a 24-h recovery period. As a result, we observed over 7,000 transformants per μg of DNA under the optimized electroporation conditions using the *R. sphaeroides* *ΔrshI* strain, which is approximately 10 times higher than that of wild-type *R. sphaeroides* under standard bacterial electroporation conditions. These findings are expected to enhance the application of *R. sphaeroides* in various industrial fields in the future.

## Introduction

Introducing plasmid DNA into a bacterial cell is an essential step in developing a recombinant strain, as it allows for further sophisticated genetic manipulations [1, 2]. Several methods are available for bacterial genetic transformation, including CaCl_2_-mediated transformation, electroporation, and conjugation [3]. Electroporation exposes bacterial cells to electrical fields, creating transient pores in the cell membranes that allows for the uptake of exogenous DNA, so that it is a widely used technique with many advantages [1, 4]. Firstly, the procedure of electroporation is simple and time-saving compared to that of conjugation [5, 6]. Electroporation is a direct transformation, whereas conjugation requires the transformation of the donor strain and culturing of both donor and recipient strains [6]. Additionally, electroporation is advantageous as it has shown reasonable efficiencies in several bacterial strains [7-9]. For example, in *Escherichia coli* strains LE392 and DH5α, transformation efficiencies as high as 10^9^ to 10^10^ transformants/μg DNA were obtained via electroporation [7]. Another study showed that using *E. coli* C strain with different types of plasmids, the transformation efficiencies were 40- to 100-times higher than those by CaCl_2_-mediated method.

*Rhodobacter sphaeroides*, chemolithoautotrophic bacteria, can be genetically engineered through conjugal DNA transfer, electroporation, and chromosomal integration, as reported in several studies [5]. However, the efficiency of electroporation as a method for introducing foreign DNA into *R. sphaeroides* can be limited due to the presence of the native *rshI* endonuclease gene, as noted in previous studies [10, 11]. The *rshI* gene encodes a restriction enzyme that recognizes and cleaves specific sites in exogenous DNA, which acts as a barrier to foreign DNA introduction [5]. Therefore, conjugative DNA transfer using specific *E. coli* strains, such as *E. coli* S17-1, has become the preferred transformation method in *R. sphaeroides* [5, 11], despite of more complication and time-consuming than electroporation. In a recent study, stepwise genetic engineering by electroporation was successfully performed to increase the expression of reaction center (RC) and light-harvesting complex 1 (LH1) proteins after deletion of the *rshI* gene in *R. sphaeroides* [10].

Bacterial transformation through electroporation is a crucial tool for genetic engineering. However, the efficiency and experimental conditions of electroporation can vary depending on the bacterial species used. The optimization of electroporation parameters, such as voltage, resistance, DNA or cell concentration, and cuvette gap interval, is essential for achieving the highest transformation efficiency for each bacterial species. In a previous study, several different species of *Lactobacilli* were subjected to identical electroporation conditions [9]. The results showed a wide range of transformation efficiencies, varying from 10^5^ CFU/μg DNA to even no transformants were observed. This suggests that the optimal electroporation conditions for each bacterial species should be carefully determined to achieve the highest transformation efficiency.

In this study, we carried out the optimization of electroporation conditions to improve the transformation efficiency in *R. sphaeroides*. At the first, *R. sphaeroides* *ΔrshI* strain was generated as a background strain for electroporation. Then, we investigated the effects of various electroporation parameters, such as the optical density of competent cells, cuvette gap interval, field strength, resistance, plasmid DNA concentration, and recovery time, on the transformation efficiency.

## Materials and Methods

### Bacterial Strain and Growth Conditions

The wild-type *R. sphaeroides* KCTC1434 was obtained from Korean Collection for Type Cultures (KCTC). *R. sphaeroides* *ΔrshI* strain was generated by knockout of the *rshI* gene, encoding the restriction endonuclease RshI, and used for the preparation of electrocompetent cells. To prepare the precultured cells, 200 μl of *R. sphaeroides* glycerol stock was added to 20 ml of Sistrom’s minimal medium in a 50 ml conical tube [12]. The cultures were incubated under light-aerobic conditions at 30°C, 150 rpm, and for 48 h.

### Construction of the *ΔrshI* Mutant

The gene fragments, including the 400 bp of the *rshI* upstream and downstream flanking regions and a kanamycin resistance gene (*Kan^R^*) with *loxP* sites, were amplified by the polymerase chain reaction (PCR) using the PrimeSTAR HS Premix (Takara, Japan) to construct the plasmid for the deletion of *rshI*. The fragments were combined through Gibson assembly and inserted into the suicide vector pSUP202pol4 [13, 14]. The constructed plasmid was transformed into *R. sphaeroides* to delete the *rshI* by homologous recombination (HR). To confirm the replacement of *rshI* into *Kan^R^*, the transformants were selected in Sistrom’s agar plate containing 50 μg/ml of kanamycin. After that, deletion of *rshI* was validated by colony PCR and sequencing. The pCM157, a broad-host-range Cre recombinase expression vector, was transferred into the selected transformants to express *cre-lox* system for antibiotic marker recycling and develop unmarked strain [15]. After validating the deletion of *rshI* and *Kan^R^* through PCR and sequencing, *R. sphaeroides* *ΔrshI* mutant was constructed.

### Transformation by Electroporation

For the preparation of electrocompetent cells, 200 μl of *R. sphaeroides* *ΔrshI* glycerol stock was added to 50 ml conical tube containing 20 ml of Sistrom’s minimal medium and incubated until an optical density (OD) from 0.5 to 2.0 was reached at 660 nm. The cells were harvested by centrifugation for 15 min at 4°C, 4,200 rpm. After discarding the supernatants, the cell pellet was washed twice using 10 ml of chilled 10% (v/v) glycerol. Afterward, the cell pellet was resuspended in 1 ml of chilled 10% (v/v) glycerol, divided into 0.1 ml aliquots, and transferred to chilled microcentrifuge tubes. The electrocompetent cells were stored at -80°C until use. The initial conditions of electroporation were established by following the conditions described in the earlier report [16]. Plasmid pBBR1MCS-2 was used during the entire optimization experiments. During the entire process, all materials were maintained in a chilled state. pBBR1MCS-2 at a concentration of 0.5-3 μg was added to the competent cells and gently mixed before transfer to a chilled electroporation cuvette with a gap size of 0.1 cm and 0.2 cm (Bio-Rad, USA) [17]. The volume of plasmid used did not exceed 10% of the volume of the competent cells. The mixture was exposed to a single pulse using a Bio-Rad Xcell Gene Pulser (Bio-Rad) with settings of 10-30 kV/cm, 25 μF, and 200-800 Ω. After the electric pulse was applied, 1 mL of Sistrom’s medium was promptly added to the cuvette and mixed gently with cell suspension. The mixture was transferred into a new chilled microcentrifuge tube and incubated with shaking at 150 rpm and 30°C for 2-24 h. To select the transformants, the cell pellets were spread on Sistrom’s agar plate containing 50 μg/ml kanamycin and incubated for 72 h at 30°C. Transformation efficiency was defined as the number of transformants per the amount of plasmid DNA.

### Statistical Analysis

All experiments were performed in triplicate and the results were expressed as mean ± standard deviation. The statistical analysis was carried out using Student’s *t*-test, and the *p*-value less than 0.05 were considered statistically significant.

## Results and Discussion

### Improvement of Electroporation Efficiency by *rshI* Deletion in *R. sphaeroides*

**Fig. 1:**
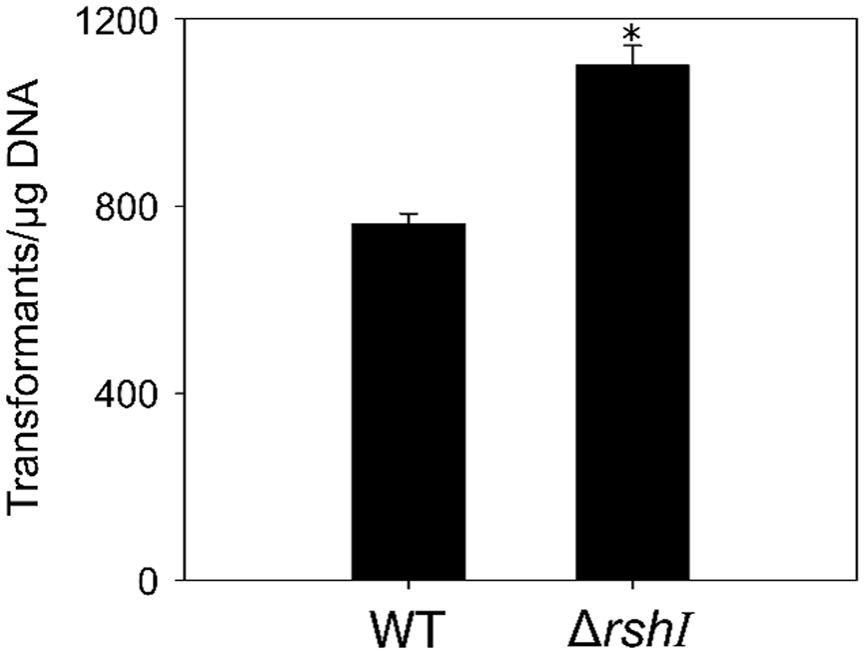
Comparison of electroporation efficiency between wild-type and *rshI* knockout mutant. The experimental parameters were determined as follows: electrocompetent cells OD_660_ of 0.5, a cuvette width of 0.1 cm, electrical parameters of 2.5 kV, 25 μF, and 400 Ω, and plasmid DNA quantity of 1 μg with 4 h recovery time. The experiments were conducted in triplicate. Error bars indicate the standard deviation of mean and the asterisk indicates statistically significant difference (*p < 0.05).

The restriction-modification systems, mediated by the restriction endonucleases, are natural defense mechanisms in microorganisms that protect against foreign DNA. These systems make the introduction of plasmid DNA difficult through traditional methods, including electroporation [18]. To facilitate the genetic manipulation of *R. sphaeroides*, we first deleted the *rshI* gene, which encodes the restriction endonuclease RshI. To eliminate the *rshI* gene via homologous recombination (HR), we constructed a cloned suicide vector, pSUP202pol4, containing a *Kan^R^* cassette flanked by loxP sites and the upstream and downstream flanking gene segments of *rshI* (Fig. 1). After transferring the engineered vector into the wild-type strain via conjugation, we confirmed the replacement of *rshI* with *Kan^R^* by selecting transformants on Sistrom agar plates containing kanamycin and performing colony PCR and sequencing. Next, we transferred the Cre recombinase expression vector pCM157 into the selected transformants to enable the expression of the *cre-lox* system for antibiotic marker recycling [15]. After validating the deletion of *rshI* and *Kan^R^* through PCR and sequencing, we generated the *R. sphaeroides* *ΔrshI* strain (Fig. S1).

To evaluate the effect of *rshI* knockout on transformation efficiency, we performed electroporation using a pBBR1MCS-2 vector in both the wild-type and *ΔrshI* strains. Electroporation was conducted with some modifications to the conditions described in an earlier report [16]. Electrocompetent cells with an OD_660_ of 0.5 and an electroporation cuvette of 0.1 cm were used. Other parameters were set 2.5 kV, 25 μF, and 400 Ω with 1 μg of DNA and a recovery time of 4 h. Transformation efficiency was indicated as the number of transformants per amount of DNA added. As depicted in Fig. 1, the results show that transformation efficiency in the *ΔrshI* strain increased by 44% compared to the wild-type. It has been reported that electroporation efficiency was similar in both the wild-type and *ΔrshI* strains when a plasmid lacking RshI recognition sites was used; however, there was a significant difference in efficiency when a plasmid containing RshI recognition sites was used. Although similar to previous results, a slight difference in electroporation efficiency was observed between the wild-type and *ΔrshI* strains when utilizing pBBR1MCS-2 containing RshI recognition sites in this study. This is reasonable because the outcome of electroporation can be influenced by various factors, such as the strain of microorganisms, electrical parameters, and the concentration of competent cells. Furthermore, previous studies have mainly relied on conjugation as a means of wild-type transformation due to the uncertain applicability of traditional transformation methods [19, 20]. Although the efficiency is still lower than in the *ΔrshI* strain, we confirmed that electroporation can be applied to the wild-type strain. Our results suggest that the deletion of the restriction endonuclease gene improved transformation efficiency and that the wild-type also has the potential for the application of traditional transformation methods.

### Optimization of Growth Conditions in Electrocompetent Cells

**Fig. 2:**
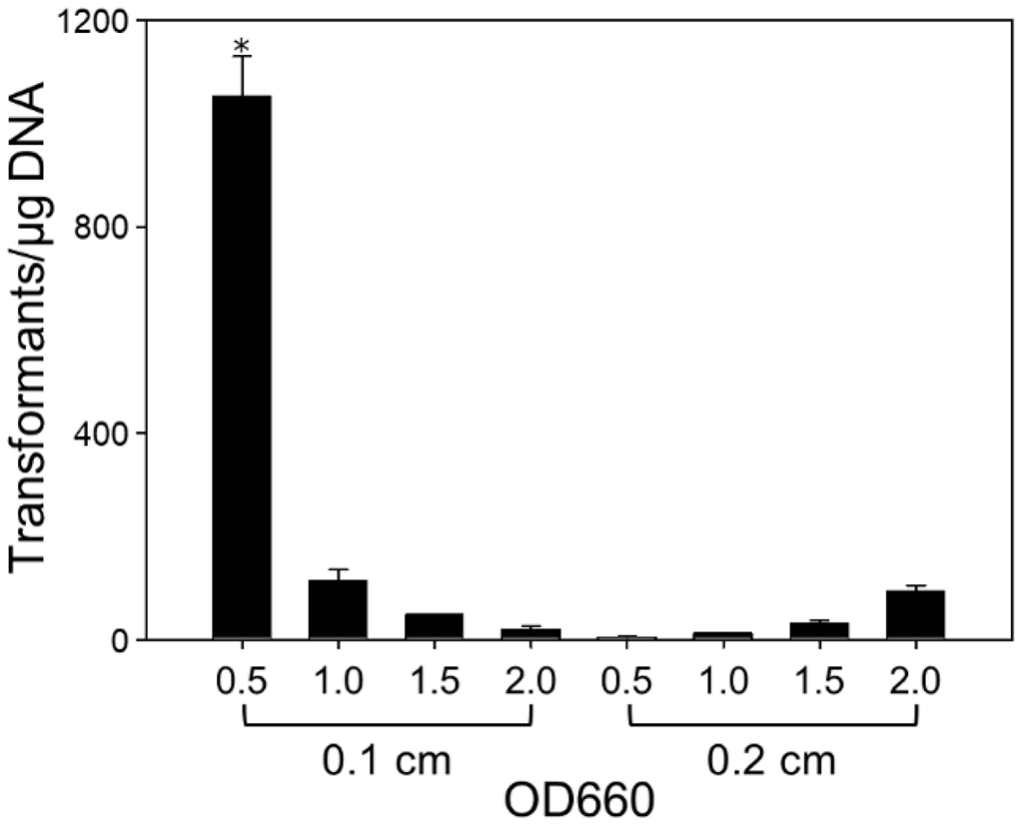
Comparison of electroporation efficiency in *R. sphaeroides* *ΔrshI* according to cell growth stage and cuvette gap sizes. The experimental parameters were determined as follows: electrical parameters of 2.5 kV, 25 μF, and 400 Ω, plasmid DNA quantity of 1 μg, and 4 h recovery time. The experiments were conducted in triplicate. Error bars indicate the standard deviation of mean and the asterisk indicates statistically significant difference (*p < 0.05).

To further enhance the transformation efficiency using the *R. sphaeroides* *ΔrshI* strain, we examined various parameters involved in the electroporation process. First, we investigated the transformation efficiency according to the growth conditions of competent cells and the gap size of the electroporation cuvette. The growth phase of competent cells was evaluated at OD_660_ ranging from 0.5 to 2.0, and electroporation cuvettes with two different gap sizes of 0.1 cm and 0.2 cm were used. Other parameters were kept consistent with those described above. Using competent cells at an OD_660_ of 0.5 and an electroporation cuvette with a gap size of 0.1 cm yielded the highest transformation efficiency (Fig. 2). These results indicate that optimizing the growth conditions of electrocompetent cells and the cuvette gap size is important for achieving high transformation efficiency. For our subsequent procedures, we used competent cells at an OD_660_ of 0.5 and an electroporation cuvette with a gap size of 0.1 cm.

Additionally, optimizing the composition of the electroporation buffer used to resuspend the electrocompetent cells is also crucial for achieving high electroporation efficiency. Utilizing a 0.2 M sucrose buffer for the electroporation of *Ralstonia eutropha* has shown better efficiency than commonly used buffers such as double-distilled water and 10% (v/v) glycerol [6]. It has been reported that the addition of sucrose and sorbitol to a 10% (v/v) glycerol buffer also helps improve efficiency in Gram-positive bacteria [21, 22]. The bacterial cell wall is considered a significant barrier to introducing foreign DNA, suggesting that increasing cell wall permeability by treating cells with wall-weakening chemicals is a potential method to further improve electroporation efficiency. Among various chemicals, including glycine, threonine, lysozyme, and penicillin, treatment with penicillin resulted in the highest increase in cell wall permeability in *Arthrobacter* [21]. Calcium chloride is also typically used for heat-shock transformation. It has been previously reported that creating electrocompetent cells after calcium chloride treatment can further increase electroporation efficiency [6]. This suggests that investigating buffer composition and chemical treatments is necessary to enhance the efficiency of *R. sphaeroides* electroporation.

### Optimization of Electroporation Parameters

**Fig. 3:**
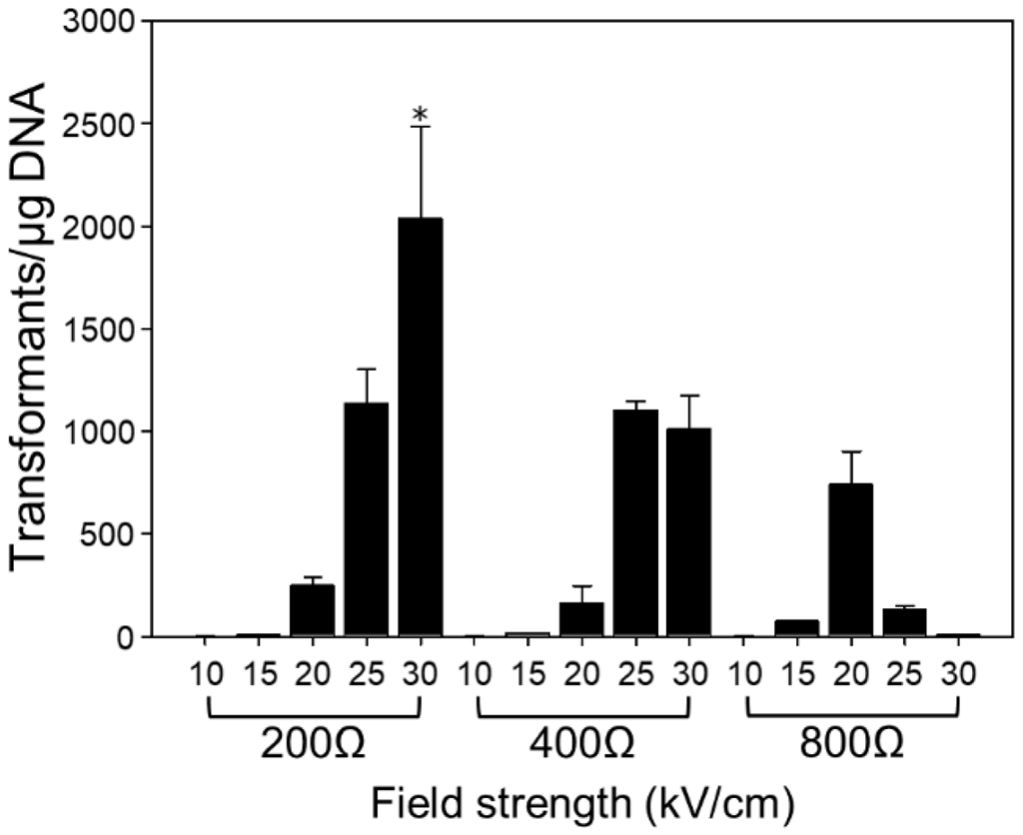
Comparison of electroporation efficiency in *R. sphaeroides* *ΔrshI* according to field strengths and resistances. The experimental parameters were determined as follows: electrocompetent cells OD_660_ of 0.5, a cuvette width of 0.1 cm, plasmid DNA quantity of 1 μg, and 4 h recovery time. The experiments were conducted in triplicate. Error bars indicate the standard deviation of mean and the asterisk indicates statistically significant difference (*p < 0.05).

The outcome of electroporation is largely dependent on electrical parameters, such as field strength (kV/cm) and resistance. Therefore, we investigated the influence of varying field strengths combined with resistance on transformation efficiency in *R. sphaeroides*. Field strengths ranging between 10 and 30 kV/cm and resistance between 200 and 800 Ω were tested. The other conditions were performed as defined in the previous section. According to the investigation of electrical parameters, the highest efficiency of electroporation was obtained with a field strength of 30 kV/cm and a resistance of 200 Ω, indicating that the efficiency was enhanced approximately 2-fold compared to the initial setting (Fig. 3). In previous studies, the optimal field strengths for lactic acid bacteria varied by species, with values of 7.5 kV/cm, 12.5 kV/cm, and 17.5 kV/cm identified as optimal for *Lactobacillus plantarum*, *Lactococcus lactis*, and *Lactobacillus buchneri*, respectively [23-25]. To achieve the greatest transformation efficiency, the optimal resistance also varies according to the microorganism. Furthermore, the electrical parameters vary not only with the species of microorganisms but also with their growth stage and morphology, indicating that optimizing electrical parameters is crucial for obtaining the best transformation efficiency [2].

**Fig. 4:**
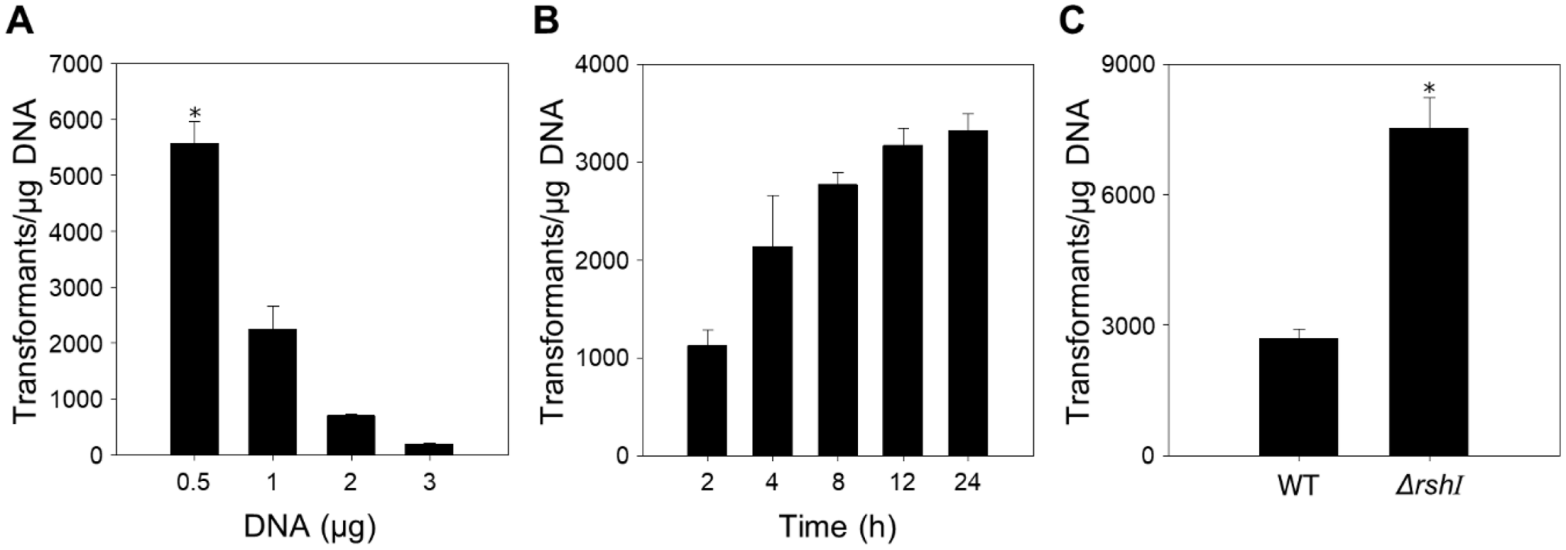
Comparison of electroporation efficiency in *R. sphaeroides* *ΔrshI* according to amounts of DNA and recovery time. (A) Effect of DNA amounts on electroporation efficiency. (B) Effect of recovery time on electroporation efficiency. (C) Comparison of electroporation efficiency in wild-type and *R. sphaeroides* *ΔrshI* under optimal conditions. The optimal parameters were determined as follows: electrocompetent cells OD_660_ of 0.5, a cuvette width of 0.1 cm, electrical parameters of 30 kV/cm, 25 μF, and 200 Ω, and plasmid DNA quantity of 0.5 μg with 24 h recovery time. The experiments were conducted in triplicate. Error bars indicate the standard deviation of mean and the asterisk indicates statistically significant difference (*p < 0.05).

Subsequently, we examined the effects of DNA amounts and recovery time on electroporation efficiency using the optimized procedure, which included competent cells at OD_660_ of 0.5, a 0.1 cm cuvette, a field strength of 30 kV/cm, and a resistance of 200 Ω. Increasing amounts of plasmid DNA from 0.5 to 3 μg were tested to evaluate their effect on electroporation in *R. sphaeroides*. The efficiency of transformation increased as the DNA quantity decreased, indicating that using 0.5 μg of plasmid DNA yielded the highest efficiency (Fig. 4A). Furthermore, recovery times ranging from 2 to 24 h were examined, with the highest efficiency observed at a recovery time of 24 h (Fig. 4B). Overall, electroporation efficiency in *R. sphaeroides* appears to increase with lower amounts of plasmid DNA and longer recovery times.

Ultimately, we optimized the electroporation conditions to achieve the highest transformation efficiency in *R. sphaeroides*. These conditions include electrocompetent cells at OD_660_ of 0.5, a cuvette width of 0.1 cm, electrical parameters of 30 kV/cm and 200 Ω, and a plasmid DNA quantity of 0.5 μg with a 24-h recovery time. Using these optimized conditions, we compared the electroporation efficiency between wild-type and *R. sphaeroides* *ΔrshI* (Fig. 4C). The transformation efficiency of wild-type and *ΔrshI* strains increased by 3.5-fold and 6.8-fold, respectively, under the optimized conditions compared to the initial conditions. Moreover, the difference in efficiency between the wild-type and *ΔrshI* strains increased by approximately 2.8-fold under the optimized conditions. These findings indicate that the optimized conditions are highly effective in enhancing transformation efficiency in *R. sphaeroides*.

Although we optimized various parameters of electroporation, challenges still remain. The optimal plasmid DNA concentration may vary depending on the type and length of the plasmid DNA. In addition, transformation efficiency can be influenced by the source and replication mode of plasmid DNA [2]. It has been reported that transformation efficiency using the same *Aeromonas* strain varies based on the type of introduced plasmid [26]. The transformation efficiency of the 4.1-kb pSDD1 plasmid was approximately 44-fold higher than that of the 10-kb pMMB67EH.Km plasmid, and the results varied greatly depending on the strain. In *Bacillus cereus*, the transformation efficiency also varied greatly depending on the size, copy number, selective marker, and replication mechanisms of the introduced plasmid [27]. When performing genetic manipulation, the length of the engineered plasmid may become longer than that of the backbone plasmid, depending on the type and number of target genes to be expressed in the host strain, suggesting that other optimized conditions may be required. In particular, the recent use of the CRISPR/Cas9 genome editing tool has expanded the spectrum of desirable plasmid traits in a variety of microorganisms, including *R. sphaeroides* [28]. We examined transformation efficiency using only the pBBR1MCS-2 plasmid, indicating that further investigation of various plasmids is necessary to fully employ genetic engineering techniques.

In this study, we optimized various parameters of electroporation to enhance efficiency in *R. sphaeroides*. Based on the results, the optimal parameters for high-efficiency electroporation in *R. sphaeroides* include electrocompetent cells at OD_660_ of 0.5, a cuvette width of 0.1 cm, electrical parameters of 30 kV/cm and 200 Ω, and a plasmid DNA quantity of 0.5 μg with a 24-h recovery time. This optimal condition is anticipated to facilitate simple and diverse genetic manipulation using *R. sphaeroides*, thereby promoting its use as an industrial platform microorganism.

## Supplemental Materials

Supplementary data for this paper are available on-line only at http://jmb.or.kr.
