# Supplementary material for: Optimization of Electroporation Conditions for Introducing Heterologous DNA into Rhodobacter sphaeroides
Source: J Microbiol Biotechnol. 2024 Sep 20;34(11):2347–52. doi: 10.4014/jmb.2408.08044 (PMC11637821; doi:10.4014/jmb.2408.08044)
Supplement: Supplementary file 1 [file jmb-34-11-2347-supple.pdf]

J. Microbiol. Biotechnol. 2024. 34(11): 2347–2352

https://doi.org/10.4014/jmb.2408.08044

Optimization of electroporation conditions for introducing heterologous DNA into *Rhodobacter sphaeroides*

Supplementary Figure

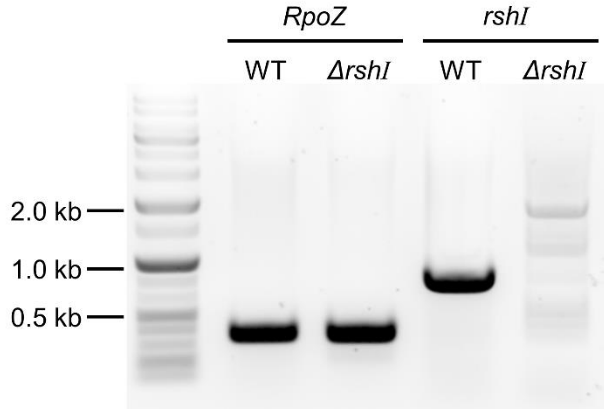

Supplementary Fig. S1. The PCR profiles of *rshI* gene knockout in wild-type and *R. Sphaereoides ΔrshI*. RpoZ gene, encoding DNA-directed RNA polymerase ω-subunit, used for the endogenous reference gene. *rshI* gene, encoding the restriction endonuclease RshI.
